# Body Composition Indices and Predicted Cardiovascular Disease Risk Profile among Urban Dwellers in Malaysia

**DOI:** 10.1155/2015/174821

**Published:** 2015-01-29

**Authors:** Tin Tin Su, Mohammadreza Amiri, Farizah Mohd Hairi, Nithiah Thangiah, Maznah Dahlui, Hazreen Abdul Majid

**Affiliations:** ^1^Centre for Population Health (CePH), Department of Social and Preventive Medicine, Faculty of Medicine, University of Malaya, 50603 Kuala Lumpur, Malaysia; ^2^Department of Development Studies, Faculty of Economics and Administration, University of Malaya, 50603 Kuala Lumpur, Malaysia

## Abstract

*Objectives*. This study aims to compare various body composition indices and their association with a predicted cardiovascular disease (CVD) risk profile in an urban population in Kuala Lumpur, Malaysia. *Methods*. A cross-sectional survey was conducted in metropolitan Kuala Lumpur, Malaysia, in 2012. Households were selected using a simple random-sampling method, and adult members were invited for medical screening. The Framingham Risk Scoring algorithm was used to predict CVD risk, which was then analyzed in association with body composition measurements, including waist circumference, waist-hip ratio, waist-height ratio, body fat percentage, and body mass index. *Results*. Altogether, 882 individuals were included in our analyses. Indices that included waist-related measurements had the strongest association with CVD risk in both genders. After adjusting for demographic and socioeconomic variables, waist-related measurements retained the strongest correlations with predicted CVD risk in males. However, body mass index, waist-height ratio, and waist circumference had the strongest correlation with CVD risk in females. *Conclusions*. The waist-related indicators of abdominal obesity are important components of CVD risk profiles. As waist-related parameters can quickly and easily be measured, they should be routinely obtained in primary care settings and population health screens in order to assess future CVD risk profiles and design appropriate interventions.

## 1. Introduction

Obesity, which refers to a state of being overweight from the excessive accumulation of body fat, often leads to a negative effect on health [1, 2]. Furthermore, obesity has been identified as a strong risk factor for cardiovascular disease (CVD) [3] and metabolic diseases such as diabetes mellitus and hypertension [4]. Several body composition indices have been used as indicators of obesity, including the body mass index (BMI), which is also considered an indicator of various health risks in general [5]. Waist circumference (WC) is another simple measurement that can assess both intra-abdominal fat mass and overall obesity [6]. Other waist-related obesity parameters include the waist-hip ratio (WHR) and the waist-height ratio (WHtR) [7]. Other risk factors for CVD (e.g., hypertension and diabetes mellitus) and lipid and glucose levels are associated with abdominal adiposity and can be assessed by these waist-related measurements [8–10]. In addition, various techniques and modern devices are available to assess body fat percentage (BF%) and body compositions, such as the whole-body dual-energy X-ray absorptiometry scan [11] and the bioelectrical impedance analysis method [12].

Future predictions of CVD incidence are essential for the development of preventive public health policies [13]. Therefore, several CVD risk prediction tools have been generated to predict ten-year CVD risk [14–22] and provide evidence-based input for CVD prevention and health system preparedness for imminent noncommunicable disease burden. Among CVD risk prediction models, the Framingham Risk Scoring (FRS) model [14] is preferred and has been widely validated [23–25] in Asian countries such as China [26], Japan [27, 28], Malaysia [29, 30], Thailand [31], Singapore, and Korea [28], and South Asians living in the United Kingdom [32]. A recently modified FRS model uses BMI rather than blood lipid levels to predict CVD risk in a primary care setting [21]. This simplified FRS model appears to be a promising tool for prediction of CVD risk in population-based screening, particularly in low- and middle-income countries that are unprepared for the current increase in CVD.

Previous studies have shown that Asian body compositions and body fat distribution differ from those of Caucasians with the same BMI, with Asians exhibiting higher BF% [33, 34]. Therefore, it is important to determine which body composition parameter correlates with a predicted CVD risk profile. The aim of the present study was to associate various body composition indices with predicted CVD risk profiles in an Asian population using the FRS model in order to estimate ten-year CVD risk among urban dwellers.

## 2. Methods

### 2.1. Study Population

Data for this study was collected from a household survey conducted within the Community Housing Projects of Metropolitan Kuala Lumpur between February and December 2012. These housing projects were developed in 2000 by the Kuala Lumpur City Hall Squatter Resettlement Program, where applicants are housed based on the following eligibility criteria: (i) married with at least one child; (ii) head of household monthly salary < MYR 2,000; and (iii) do not own any property within 35 km of Kuala Lumpur. Households from four Community Housing Projects were selected using a simple random-sampling method.

Adults of selected households were invited for medical screening, which included anthropometric measurements and blood sampling conducted by trained research assistants and nurses under the supervision of a medical doctor. Individuals with incidence of CVD (coronary death, myocardial infarction, coronary insufficiency, angina, ischemic stroke, hemorrhagic stroke, transient ischemic attack, peripheral artery disease, or heart failure) were excluded from the study. Written consent was obtained from respondents. Ethics application was approved by University of Malaya Medical Centre Medical Ethics Committee (MEC ref. number 890.161).

### 2.2. Sample Size Determination

We estimated the sample size using G^*^Power software (version 3.1.9.2) [35]. As no published evidence on the correlation of body composition with predicted CVD risk was available, we used different correlation coefficients (0.1–0.9) to estimate the sample size. A calculated sample size of 751 participants was estimated to provide 80% power and type I error of 0.05.

### 2.3. Measurements

Height was measured to the nearest 0.1 cm by a stadiometer (SECA 217; Seca, Hamburg, Germany), and weight was measured to the nearest 0.1 kg (SECA 813 Digital High Capacity Floor Scale; Seca). BMI was calculated as weight over squared height (kg/m^2^). BF% was assessed by bioelectrical impedance analysis using a body composition analyzer (SC-240 MA; Tanita Corp, Tokyo, Japan). Accuracy of this device has been validated in the USA by comparing it with dual-energy X-ray absorptiometry [36] and is comparable to results from the InBody370 body composition analyzer [37]. We used the single measurement as has been used in previous studies [37, 38]. Stretch-resistant tape (SECA 201; Seca) was positioned midway between the lowest rib margin and the iliac crest to measure WC, and the widest point of the hip/proximal thigh, just below the gluteal fold, was used to obtain the hip circumference; measurements were to the nearest 1 mm. WHR and WHtR were derived by dividing WC to hip circumference and height, respectively.

The mean arterial blood pressure was calculated from two measurements (Omron HEM 7211; Omron Corp., Kyoto, Japan) taken from the left arm of participants in a seated position. A registered nurse collected blood samples and blood sugar and lipid profiles were assessed on a Dimension Vista 1500 Intelligent Lab System (Siemens, Munich, Bavaria, Germany) in a certified laboratory of a tertiary hospital. Individuals with a nonfasting random blood sugar ≥11.0 mmol/L and/or were under diabetic treatment were considered as diabetic [39, 40].

### 2.4. CVD Risk Profile

Ten-year CVD risk profiles were estimated using the laboratory-based FRS model [21]. This model has been validated in Malaysia with 600 patients attending the Family Medicine Clinic of the tertiary hospital. The authors concluded that CVD events predicted by FRS were only marginally higher than observed [29, 30]. This model incorporates age (in years), total and high-density lipoprotein cholesterol levels, systolic blood pressure, antihypertensive medication use (obtained from the questionnaire), smoking status (≥1 cigarette per day), and diabetic status. The CVD risk profile was assessed by summing the FRS points derived from each individual's variables converting them into a CVD risk percentage. The general cardiovascular risk prediction charts and detailed discussion can be found in D'Agostino Sr. et al. [21].

### 2.5. Demographic and Socioeconomic Variables

Demographic and socioeconomic information were collected, such as age, gender, ethnicity/race, marital status, highest education level (none = 0 y, primary = 1–6 y, secondary = 7–12 y, or tertiary ≥13 y of education), monthly income level (<1000, 1000–1999, 2000–2999, and ≥3000 MYR), and occupational status (e.g., paid-employee, self-employed, inactive [including retired], homemaker, student, and traineeship or apprenticeship [41, 42]).

### 2.6. Statistical Analysis

All statistical analyses were performed using Stata v11.2 software (StataCorp, College Station, TX, USA). The demographic and socioeconomic characteristics are presented as a percentage, and measured indices are presented as mean ± standard deviation. Differences between and among categories were assessed with Student's* t*-tests and analyses of variance. Pearson correlation analyses were used to evaluate associations of body composition indices with the predicted CVD risk. Correlation coefficients were adjusted for demographic and socioeconomic variables (adjusted correlation analysis). For all analyses, *P* < 0.05 was considered as statistically significant.

## 3. Results

We invited 2360 adults (≥18 years old) from 833 selected households to participate in anthropometric measurements and blood sampling; the response rate was 50.5%. There was no difference between respondents and nonrespondents with respect to age, gender, or ethnicity. To evaluate the ten-year CVD risk profile, respondents <30 y and who had ≥1 CVD incidence were excluded. A total of 882 respondents with a mean age of 48.1 ± 11.7 y were included in the analyses.

Table 1 shows the characteristics of the sample population and the mean of predicted CVD risk according to demographic and socioeconomic variables. Males had significantly higher CVD risk compared to females. In addition, significant differences in CVD risk were observed according to age, education, occupation, and marital status (all *P* < 0.05). However, there was no ethnic difference in predicted CVD risk.

The correlation analysis (Table 2) illustrated significant associations between body composition parameters and predicted CVD risk. In both males and females, CVD risk was most strongly correlated to WHR and WHtR (all *P* < 0.001). In addition, WC was strongly correlated with CVD risk in both males and females, as was BF%, though to a lesser extent.

CVD risk was also assessed according to body composition measures after adjusting for age, ethnicity, marital status, education level, occupation status, and income level, as well as by gender (Table 3). In males, WHR had the strongest correlation with ten-year CVD risk, followed by WC and WHtR, whereas BMI had the strongest association in females, followed by WHtR and WC (all *P* < 0.001). Overall, WC had the strongest correlation with CVD risk, followed by WHtR and BMI (all *P* < 0.001).

## 4. Discussion

The results of this study demonstrate that waist-related indicators of abdominal obesity are significant risk factors for CVD. These indices (i.e., WHR, WHtR, and WC) were strongly associated with cardiovascular risk in both genders. In various nationally representative studies conducted in Malaysia, Malays were shown to have the highest prevalence of CVD risk factors compared to other ethnic groups [43–45], and those residing in urban areas were more likely than other ethnicities to experience one of the CVD risk factors [46]. In contrast, our results indicate that in the next ten years, the risk of CVD might not differ among ethnic groups in urban Malaysia.

Our findings in men are in line with previous studies in which WHR was a reliable factor for evaluating chronic disease and CVD risk [47, 48] and long-term CHD and CVD incidence [2, 49–52]. In addition, a previous study claimed that waist-related indices are useful for predicting CVD risk and life-style determinants [53]. Several other studies have suggested that WHtR is the strongest anthropometric indicator for development of CVD or coronary heart disease (CHD) [54–56]. Furthermore, a study in Taiwan found that WHtR had the strongest correlation with CVD risk factors compared to other indices [57]. This is supported by a meta-analysis showing that WHtR has the strongest relationship with CVD risk factors, particularly with obesity, hypertension, and dyslipidemia, compared to other body composition measurements [47]. In addition, WHR and WC had stronger associations with predicted risk of myocardial infarction than BMI [2, 58, 59].

The simplest anthropometric index, WC, was one of the body composition measurements that was most strongly associated with risk in both pre- and postadjusted correlation analyses, consistent with previous findings of CVD [60, 61] and CHD [54, 55]. However, in contrast to the present results, other studies found that WC was stronger than WHR and BMI in women [62, 63] and has direct effects on long-term CHD and CVD [2, 49–52].

The association of BMI with vascular diseases is varied. Wilson et al. [1] concluded that higher BMI levels can be considered as a risk determinant for CHD. Other studies found direct associations between high BMIs and incidence and mortality of CHD in both genders [1, 2, 50, 52, 64]. However, in our study, there was a weak correlation between BMI and CVD in men, but BMI was associated with CVD risk in females, which is also in line with previous studies conducted in Canada [60]. However, the PROCAM study found that the BMI by itself did not contribute to CVD risk but rather may mediate the effect of other risk factors [52].

BF% has been considered the best estimate of an individual's body fat and future risk of CVD [65, 66]. In our analyses, however, BF% was only weakly associated with predicted CVD risk compared to other body composition indices. Another previous study also found that BMI and WHR were better predictors of CVD risk than BF% [67]. It is possible that BF% is only an important contributor to CVD risk in obese individuals [68].

## 5. Conclusion 

This study demonstrates that waist-related body composition indices are associated with predicted CVD risk among urban dwellers in Malaysia. As these parameters are simple and quick to obtain, they should be routinely recorded in the primary care setting and in population-based health screening in order to assess future CVD risk and design appropriate interventions.

## Figures and Tables

**Table 1:** Characteristics and predicted cardiovascular disease (CVD) risk (*n* = 882).

Characteristic	Value	Predicted CVD risk mean (95% confidence interval)
Age, y		
30 to 39	228 (22.85)	3.77 (3.31–4.24)
40 to 49	287 (32.54)	9.01 (8.16–9.86)
50 to 59	209 (23.70)	15.50 (14.25–16.76)
≥60	158 (17.91)	21.00 (19.62–22.38)
*F*-test (prob.)		201.08^**^
Gender		
Male	374 (42.40)	15.59 (14.59–16.60)
Female	508 (57.60)	8.21 (7.51–8.91)
*t*-test (prob.)		12.21^**^
Ethnicity		
Malaysian	723 (81.97)	11.09 (10.41–11.78)
Indian	144 (16.33)	12.36 (10.62–14.10)
Chinese/Others	15 (1.70)	13.62 (7.85–19.39)
*F*-test (prob.)		1.48
Education		
None	70 (8.16)	15.65 (13.14–18.16)
Primary	195 (22.73)	14.25 (12.83–15.68)
Secondary	551 (64.22)	9.70 (8.98–10.42)
Tertiary	42 (4.90)	6.00 (3.82–8.19)
*F*-test (prob.)		22.67^**^
Income, MYR		
<1000	168 (19.05)	12.52 (10.96–14.08)
1000–1999	352 (39.91)	10.73 (9.75–11.71)
2000–2999	205 (23.24)	10.55 (9.30–11.80)
≥3000	133 (15.08)	10.76 (9.28–12.24)
*F*-test (prob.)		1.76
Occupation		
Paid-employee	332 (38.69)	9.16 (8.28–10.04)
Self-employed	131 (15.27)	13.44 (11.68–15.21)
Homemaker	223 (25.99)	7.40 (6.49–8.31)
Other	126 (14.69)	16.07 (14.28–17.86)
Inactive	46 (5.36)	21.65 (19.18–24.12)
*F*-test (prob.)		45.53^**^
Marital status		
Married	659 (76.81)	8.29 (6.47–10.11)
Divorced	71 (8.28)	11.04 (10.32–11.76)
Widow/widower	55 (6.41)	11.90 (9.64–14.15)
Single	73 (8.51)	13.61 (10.82–16.41)
*F*-test (prob.)		3.71^*^
Hypertension	421 (47.8)	
Diabetes	157 (17.8)	
Smoking	153 (17.4)	
Body mass index	27.1 (5.8)	
Body fat percentage	32.4 ± 10.8
Waist-hip ratio	0.9 ± 0.1	
Waist-height ratio	0.6 ± 0.1	
Waist circumference, cm	88.6 ± 12.5	

Data are resented as mean ± standard deviation or as *n* (%); ^*^ *P* < 0.05; ^**^ *P* < 0.001.

**Table 2:** Correlations between ten-year cardiovascular disease (CVD) risk and body composition measures.

Measure	Predicted CVD risk					
	Males		Females		Total	
	*r*	*P*	*r*	*P*	*r*	*P*
BMI	0.0639	0.2197	0.1686	<0.001	0.0377	0.2639
BF%	0.1366	<0.01	0.1749	<0.001	−0.1504	<0.001
WHR	0.4321	<0.001	0.2769	<0.001	0.4356	<0.001
WHtR	0.3133	<0.001	0.3540	<0.001	0.2295	<0.001
WC	0.2753	<0.001	0.2664	<0.001	0.2795	<0.001

BF% = body fat percentage; BMI = body mass index; WC = waist circumference; WHR = waist-hip ratio; WHtR = waist-height ratio.

**Table 3:** Adjusted correlations between ten-year cardiovascular disease (CVD) risk and body composition measures.

Measure	Predicted CVD risk					
	Males^1^		Females^1^		Total^2^	
	*r*	*P*	*r*	*P*	*r*	*P*
BMI	0.1281	<0.05	0.2715	<0.001	0.2037	<0.001
BF%	0.1310	<0.05	0.2257	<0.001	0.1842	<0.001
WHR	0.2217	<0.001	0.1119	<0.05	0.1602	<0.001
WHtR	0.1816	<0.001	0.2682	<0.001	0.2180	<0.001
WC	0.2011	<0.001	0.2655	<0.001	0.2331	<0.001

BF% = body fat percentage; BMI = body mass index; WC = waist circumference; WHR = waist-hip ratio; WHtR = waist-height ratio.

^1^Adjusted for age, ethnicity, occupation, marital status, income level, and education level; ^2^adjusted for all plus gender.

## Abbreviations

BF%: Body fat percentage
BMI: Body mass index
CHD: Coronary heart disease
CVD: Cardiovascular disease
FRS: Framingham Risk Scoring model
WC: Waist circumference
WHR: Waist-hip ratio
WHtR: Waist-height ratio.
